# Complete mitogenome sequence of *Aedes* (Stegomyia) *aegypti* derived from field isolates from California and South Africa

**DOI:** 10.1080/23802359.2018.1495117

**Published:** 2018-10-03

**Authors:** Hanno Schmidt, Mark J. Hanemaaijer, Anthony J. Cornel, Gregory C. Lanzaro, Leo Braack, Yoosook Lee

**Affiliations:** a Vector Genetics Laboratory, Department of Pathology, Microbiology, and Immunology, School of Veterinary Medicine, University of California–Davis, Davis, CA, USA;; b Department of Entomology and Nematology, University of California–Davis, Davis, CA, USA;; c UP Institute for Sustainable Malaria Control and MRC Collaborating Centre for Malaria Research, Faculty of Health Sciences, University of Pretoria, Pretoria, South Africa

**Keywords:** Arbovirus vector, California, South Africa, mosquito

## Abstract

The *Aedes aegypti* mitogenome (Mt) sequences of field isolates from California and South Africa revealed a deletion between position 14,522 and 14,659 of the Mt contig of the AaegL5 reference genome. The length of the mitogenome of the California isolate was 16,659 bp and had 99.0% similarity with the AaegL5 Mt contig. The South African isolate sequence was 16,600 bp long and had 97.9% similarity with the reference. The region between 1496 and 1664 bp is similar to a nuclear pseudogene that might be a copy of a portion of the mitochondrial genome.

*Aedes aegypti* (Linnaeus), also known as the yellow fever mosquito, vectors multiple human arboviral diseases that include yellow fever, dengue fever, and Zika fever. This species originated in Africa (Nelson 1986; Mousson et al. 2005) but has now spread around the globe (Gloria-Soria et al. 2014; Akiner et al. 2016; Cornel et al. 2016). The fully-assembled genome of *A. aegypti* (AaegL5) including mitogenome (Mt) became available in 2017 (Matthews et al. 2017). Here we report two complete mitogenome sequences of wild caught *A. aegypti*—one from Clovis, California (36.813°N, 119.667°W), USA and the other from the Kruger National Park, South Africa (23.116°S, 31.430°E).

DNA extraction and library preparation were conducted using the protocol described by Nieman et al. (2015) and Yamasaki et al. (2016). The libraries were sequenced for 150 bp paired-end reads using a HiSeq 4000 instrument at UC Davis. Raw sequencing reads were trimmed using Trimmomatic version 0.36 (Bolger et al. 2014). Mt contigs were assembled for each individual using NOVOPlasty version 2.6.7 (Dierckxsens et al. 2017). Resulting contigs from the two mosquitoes contained a deletion of 138 bp in the 12S rRNA region starting from position 14,522 of the AaegL5 Mt, thereby resembling the state in other Culicidae (data not shown). The length of the California isolate Mt (Genbank: MH348176) was 16,659 bp and had 99.0% sequence similarity with AaegL5 Mt. The South African isolate Mt (Genbank: MH348177) was 16,600 bp and had 97.9% sequence similarity with the reference. A phylogeny including other vector species is shown in Figure 1. DNA samples are kept in Vector Genetics Laboratory at UC Davis.

Mitochondrial pseudogenes are known to be prevalent in the *A. aegypti* nuclear genome (Hlaing et al. 2009), which may influence mapping. To compare the mapping quality, trimmed reads were mapped to references using BWA (Li 2013) with default settings. Mapping performance was compared and inspected visually using IGV version 2.4.10. Competitive mapping together with the AaegL5 nuclear genome resulted in lower coverage on the mitogenome (mean 853X) than noncompetitive mapping (mapping to mitogenome only; mean 1,486X). This indicates that some mitochondrial reads were mapped to nuclear contigs rather than the mitogenome during competitive mapping, potentially increasing the bias in nuclear genome genotype calls. In noncompetitive mapping, a portion of COX1 between 1,496 and 1,703 bp had 40% increase in coverage due to nuclear pseudogene reads mapped to mitogenome. For nuclear genome analysis of *A. aegypti*, we recommend mapping the sequences to the Mt first to filter out Mt reads and then use the remaining unmapped reads to map to the nuclear genome.

**Figure 1.**
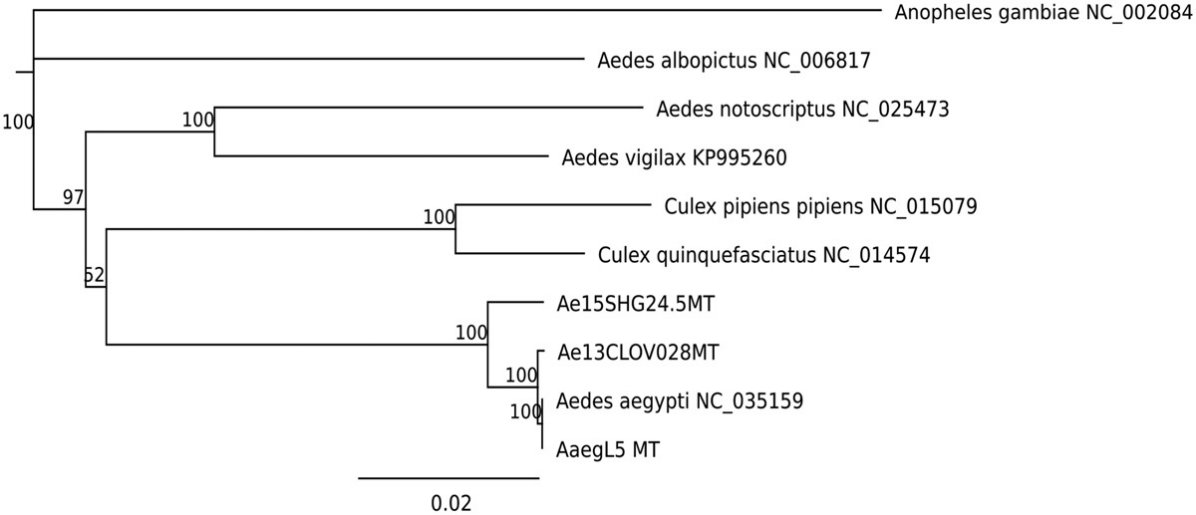
Phylogenetic tree based on mitochondrial genome sequences of mosquito species. Genbank IDs used in this analysis are provided next to each species name. Jukes–Cantor model was used to calculate pairwise genetic distances. Neighbor–Joining method was used to build this tree. Numbers at nodes indicates bootstrap values out of 200 replicates. *Anopheles gambiae* was used as an outgroup.AQ1: Please confirm the corresponding author’s address and correct if it is inaccurate.

In the revised Mt sequence, the gap in mapping coverage is removed. The gap was confirmed by mapping three datasets from the NCBI Sequence Read Archive onto AaegL5 (SRR6063610, SRR6063610, SRR871497). Additionally, we confirmed the exact position of the deletion by perfect string matching of 40-mers. Therefore, the revised versions of the mitochondrial genome seem to represent a pattern broadly displayed within *A. aegypti*. AaegL5 was sequenced from the Liverpool strain, a colony strain for >80 years that might show specific characteristics.
